# Comparative analysis of village doctors’ relative deprivation: based on two cross-sectional surveys

**DOI:** 10.1186/s12875-024-02385-6

**Published:** 2024-04-25

**Authors:** Qiusha Li, Zixuan Zhao, Chunxiao Yang, Bei Lu, Chenxiao Yang, Jiahui Qiao, Dongmei Huang, Zhongming Chen, Wenqiang Yin

**Affiliations:** 1 School of Public Health, Shandong Second Medical University, Weifang, Shandong China; 2 School of Management, Shandong Second Medical University, Weifang, Shandong China

**Keywords:** Primary health care, Rural health, Village doctors, Relative deprivation

## Abstract

**Background:**

Village doctors are the main health service providers in China’s rural areas. Compared with other rural groups, they will have a sense of relative deprivation, which has an impact on their practice mentality and job stability. This study aims to analyze the changes and causes of relative deprivation among village doctors, so as to improve the stability of them.

**Methods:**

The data were collected from two surveys conducted in Shandong Province in 2015 and 2021. In 2015, 322 village doctors were surveyed and 307 questionnaires were collected, with a recovery rate of 95.3%. In 2021, 394 village doctors were surveyed and 366 questionnaires were collected, with a recovery rate of 92.9%. Descriptive and univariate analysis were used to compare the changes before and after the survey.

**Results:**

The scores of vertical deprivation of village doctors increased from 2.77 ± 0.81 in 2015 to 3.04 ± 0.83 in 2021, with a statistically significant difference (*P* < 0.001). The reference group selected by village doctors changed from village teachers to ordinary villagers. Compared to village teachers, the horizontal deprivation score of village doctors increased from 3.47 ± 0.87 to 3.97 ± 0.77, with a statistically significant difference (*P* < 0.001). Compared to villagers, only the professional reputation deprivation score increased, from 2.38 ± 0.93 to 2.68 ± 0.76, with a statistically significant difference (*P* < 0.05).

**Conclusions:**

As time goes by, village doctors fail to reach the expected level in terms of economic income, social status, professional reputation and living standards, resulting in a sense of relative deprivation. This may have a negative impact on village doctors’ work motivation and behavior, and will fail to guarantee the sustainability of the team. We should pay attention to this unbalanced mentality of village doctors.

## Introduction

Village doctors are the providers of medical and health services in rural China, which originated from the “barefoot doctors” in the early days of the founding of the People’s Republic of China. Most barefoot doctors have not received professional medical education and formal training, they have only received a minimum of basic medical and paramedical training [1]. However, as a product of China’s specific historical period, they have greatly alleviated the shortage of medical resources in rural areas [2]. The “barefoot doctor” was once recognized by the World Health Organization (WHO) as a successful model for providing primary health care in developing regions with inadequate medical resources. In 1985, the Ministry of Health decided to stop using the term “barefoot doctor”. This group was transformed into “village doctors” through a system of examination and elimination [3]. In order to standardize the practice of village doctors, the current village doctors must pass the examination and obtain the corresponding certificate to carry out medical work. They are responsible for the two functions of basic medical service and basic public health service. Their daily work includes reception, diagnosis, treatment and care [4].

According to the National Bureau of Statistics, by the end of 2020, there were 609,000 village clinics in 509,000 administrative villages in China, with about 747,000 village doctors. They are responsible for the medical and health services of 509.79 million village people and serving as the gatekeepers of the health of village residents [5]. After the outbreak of COVID-19, village doctors, as the main providers of primary medical and health services in village areas, have played an important role in epidemic prevention and control. In 2020, the number of visits to village clinics reached 1.43 billion, with an average of 2349 visits per village clinic [5]. On the one hand, they continue to provide population-wide and patient-centered care for rural residents, for example, maintaining contact with patients with chronic diseases to ensure continuity of treatment [6]. More importantly, village doctors have taken on new responsibilities and work content during the pandemic, including screening and reporting fever patients, actively arranging migrant returnees, conducting health monitoring of home-isolated people, administering COVID-19 vaccines, nucleic acid testing, etc., in order to prevent the spread of the epidemic in village areas [7].

As the main force of health services in rural areas, the practice mentality of village doctors is also the focus of academic research. However, few scholars have paid attention to the sense of relative deprivation of village doctors in social comparison. Relative deprivation is a subjective cognitive and emotional experience in which individuals perceive themselves as inferior in comparison, and then experience negative emotions such as dissatisfaction and anger [8]. It was first proposed by Stouffer [9], an American sociologist, in 1949, and then Merton [10] expanded the relevant research to form the reference group theory. Gurr [11] believed that the psychological process of relative deprivation is social comparison, which originated from the reference group and people’s past experience. The sense of relative deprivation generated when people are compared with the reference group with similar but not identical social status is called horizontal relative deprivation, while the sense of relative deprivation generated when they fail to achieve the expected goal compared with their past self is called vertical relative deprivation [12–15]. It will have a negative impact on employees’ professional mentality and work behavior, such as lack of interest in work, reduced enthusiasm, inefficiency and so on [16, 17]. It can also adversely affect the physical and mental health of individuals [18–20].

Village doctors work and live in rural society, and they are often compared with other rural social groups [21]. The sense of relative deprivation of rural doctors is the psychological reaction to some absolute or relative differences after comparison. Recently, the collective resignation of rural doctors and other incidents to a certain extent are also due to the existence of a sense of relative deprivation, which leads to changes in professional mentality and behavior [8]. The sense of relative deprivation caused by this comparison has a negative impact on the professional mentality of rural doctors, and this adverse psychological state will lead to the reduction of rural doctors’ satisfaction with the health care reform and affect the realization of the reform goals.

The existing research pays little attention to the relative deprivation mentality of village doctors. The research mainly focuses on village doctors’ job satisfaction, job stability and job burnout status and influencing factors, such as Lennon [22] and Zhang’s [23] study on village doctors’ job satisfaction, Hain [24] and Zhang’s [25] study on job burnout status and influencing factors. Lack of attention to the individual work motivation of village doctors, and lack of longitudinal tracking to compare the changes of village doctors’ practice mentality. Therefore, to bridge these gaps and add more evidence on individual-level determinants in primary health care. This study aims to make a theoretical contribution to the limited literature on the relative deprivation of village doctors through a repeated cross-sectional survey. At the same time, we hope to provide a basis for improving the practice mentality of village doctors and stabilizing the rural medical team. We refer to the theory of relative deprivation and the studies of Fu [26], Séamus [27] and others, divides the village doctors’ relative deprivation into horizontal relative deprivation and vertical relative deprivation. Horizontal relative deprivation refers to the psychological gap between rural doctors and other rural social groups in terms of income, status, reputation and living standards. Vertical relative deprivation refers to the psychological gap between rural doctors and their “past selves” when they fail to reach their expected level in the process of development. In addition, we based on the data of two surveys in Shandong Province in 2015 and 2021, to analyze the changes of village doctors’ relative deprivation before and after the COVID-19 epidemic, and explore the possible reasons.

## Methods

### Study design

The study was mainly conducted in Shandong Province, and the respondents were village doctors. Shandong Province, located in the eastern part of China, is a major agricultural and populous province in China, where the rural population accounts for 36.95% of the total population [2]. By the end of 2020, Shandong Province has a total of village clinics 53,523, rural doctors 80,134, rural health human resources are relatively rich [28]. The data were collected from two surveys in 2015 and 2021.

In 2015, we conducted a baseline survey using multi-stage stratified random sampling. First, according to the level of economic development, we choose two prefecture-level cities, W and L, which are both medium economic areas in Shandong Province. Second, three counties (cities and districts) with different economic levels were randomly selected from each prefecture-level city, three townships were randomly selected from each county. Finally, six village clinics were randomly selected from each township. All the on-the-job village doctors were included in the survey site. A total of 322 village doctors were surveyed. In 2021, the survey of village doctors in these two cities was continued according to the same sampling method, and the sample size collected was slightly higher than that in 2015 due to the increase in the number of village doctors in each village clinic as a result of the policy requirement to integrate village clinics. A total of 394 village doctors were surveyed.

To protect the privacy of the respondents, the questionnaires were filled out anonymously by individuals. In order to ensure the quality of the questionnaire, during the data collection stage, a survey team was formed by teachers and doctoral/postgraduate students of health service management, to ensure effective communication with village doctors. Before the survey, they were trained intensively, including the survey topic, process, questionnaire content explanation, and precautions, etc. We assigned 3–5 investigators to each site, with a total of 12 investigators in 2015 and 16 in 2021. In the process of investigation, investigators will answer questions in real time to avoid problems such as wrong filling and missing filling. At the end of the survey, each investigator needs to check the questionnaires in his hands, and then check all the questionnaires collected by the survey team. Invalid questionnaires such as too many missed answers, logical errors, skipping answers errors and continuous selection of the same option were eliminated. Finally, 307 valid questionnaires (2015) and 366 valid questionnaires (2021) were collected, with effective recovery rates of 95.3% and 92.9%, respectively. The questionnaire was administered in Chinese.

### Measurement

#### Social demographic characteristics.

The socio-demographic characteristics in the questionnaire included gender (male, female), age (≤ 30, 31–40, 41–50, ≥ 51), education level (junior high school and below, secondary technical school graduates, junior college degree and higher), professional qualification (assistant medical practitioners, medical practitioners and practice qualifications of rural doctors). In terms of qualifications, only those with bachelor’s degree or above can apply for the qualification of medical practitioners. Assistant medical practitioners only need secondary school or junior college education, and the difficulty of the examination is relatively small. However, assistant medical practitioners can only practice in township hospitals or village clinics after registration [29]. The rural doctor’s practice qualifications refers to the rural doctor’s certificate that the former “barefoot doctor” can only obtain after passing the examination [1].

#### Measurement of relative deprivation.

Referring to the research of Qin [21], Xu [30], Hilda [31] and Heather [32], we designed the “Relative Deprivation Scale”, which combined the characteristics of village doctors, measured their relative deprivation compared with the reference object horizontally and vertically. The detailed content of this scale can be referred to Tang [8] and Chen’s [33] study. It mainly includes three parts: ①"The most frequently compared population”. There is one question in this part, asking the village doctors often compared with which population. The options included village managers (township cadres, village cadres), doctors in township hospitals, villagers of the same age, other village doctors and rural teachers [34]. Rural teachers refer to those who work in rural schools. Since the founding of New China, most of the population has lived in rural areas. Rural teachers have played an important role in improving the cultural quality of the rural population and are the key to the development of rural education [35]. ②Horizontal relative deprivation, that is “comparison with the belonging group”. This part contains four items: economic income, social status, professional reputation and living standard. ③Vertical relative deprivation, that is “comparison with one’s own past”, the entry is the same as above. The Likert five-point scale was adopted for all options, and 1–5 means a lot of improvement, a little improvement, about the same, a little reduction and a lot of reduction in turn.

In terms of the structural validity of the questionnaire, the KMO (Kaiser Meyer Olkin) of the horizontal relative deprivation questionnaire of village doctors in 2015 was 0.77, and the Bartelett’s sphericity test χ^2^ = 471.74, *df* = 6, *P* < 0.001, which was suitable for factor analysis. The construct validity of the questionnaire was analyzed by principal component analysis and maximum variance orthogonal rotation, and one common factor was extracted, namely horizontal relative deprivation. The cumulative variance contribution value of the common factor was 65.21%, and the load value of each factor was between 0.66 and 0.86. Vertical relative deprivation questionnaire KMO = 0.71, Bartelett’s sphericity test χ^2^ = 324.54, *df* = 6, *P* < 0.001. One common factor was extracted, namely vertical relative deprivation, and the cumulative variance contribution value was 58.93%, and the load value of each factor was between 0.68 and 0.81. The KMO of village doctors’ horizontal relative deprivation questionnaire in 2021 was 0.74, and the Bartelett’s sphericity test χ^2^ = 914.58, *df* = 6, *P* < 0.001. One common factor was extracted, namely horizontal relative deprivation. The cumulative variance contribution value was 73.73%, and the load value of each factor was between 0.83 and 0.90. Vertical relative deprivation questionnaire KMO = 0.77, Bartelett’s sphericity test χ^2^ = 787.74, *df* = 6, *P* < 0.001. One common factor was extracted, namely vertical relative deprivation, the cumulative variance contribution value was 72.38%, and the load value of each factor was between 0.66 and 0.79. The above shows that the construct validity of the questionnaire is good. In terms of questionnaire reliability, the Cronbach’s *α* of the horizontal relative deprivation questionnaire for village doctors in 2015 was 0.819, and the Cronbach’s *α* of the vertical relative deprivation questionnaire was 0.762. In 2021, the Cronbach’s *α* of the horizontal relative deprivation questionnaire was 0.880, and the Cronbach’s *α* of the vertical relative deprivation questionnaire was 0.870, indicating that the questionnaire had good reliability.

### Statistical analysis

SPSS version 21 (IBM Corp, Armonk, NY, USA) was used to set up the database and perform statistical analysis. Because of its ease of use, flexibility and scalability, the software has been widely used in the existing research. Firstly, the software was used to make a descriptive analysis of the basic demographic characteristics and the current situation of relative deprivation of the respondents. The count data were described by frequency and proportion, and the measurement data were described by mean and standard deviation. Secondly, the chi-square test was used to compare whether there were differences between the two surveys populations. T-test was used to compare the differences between the two surveys. The significance level was set at *P* < 0.05.

## Results

### Demographic characteristics

The basic characteristics of the respondents in 2015 and 2021 are shown in Table 1. In 2015, the effective recovery rate of the questionnaire was 95.3%, a total of 307 village doctors were surveyed, including 143 in City W and 164 in City L. In 2021, the effective recovery rate of the questionnaire was 92.9%, a total of 366 village doctors were surveyed, including 158 in City W and 208 in City L. Most of the respondents in the two surveys were male. In 2015, the respondents were between 31 and 40 years old, and in 2021, the respondents were between 41 and 50 years old. They were mainly educated in secondary technical school, and most of them had practice qualifications of rural doctors.

**Table 1:** Demographic characteristics of village in two surveys

Characteristics	2015(*N* = 307) N(%)	2021(*N* = 366) N(%)	χ^2^	*P*
**Region**				
City W	143(46.6)	158(43.2)	0.785	0.392
City L	164(53.4)	208(56.8)		
**Gender**				
Male	225(73.3)	248(67.8)	2.445	0.128
Female	82(26.7)	118(32.2)		
**Age**				
≤ 30	26(8.5)	7(1.9)	51.806	<0.001
31~	118(38.4)	84(23.0)		
41~	83(27.0)	186(50.8)		
51~	80(26.1)	89(24.3)		
**Educational background**				
Junior high school and below	26(8.5)	6(1.6)	35.345	<0.001
Secondary technical school graduates	217(70.7)	221(60.4)		
Junior college degree and higher	64(20.8)	139(38.0)		
**Practice qualification**				
Assistant medical practitioner	17(5.5)	91(24.9)	52.181	<0.001
Medical practitioner	24(7.8)	32(8.7)		
Practice qualifications of rural doctors	255(83.1)	231(63.1)		

### Changes in the selection of reference groups of village doctors

In the 2015 survey, rural teachers were the most reference group that chosen by rural doctors, accounting for 59.3%, followed by villagers (16.9%) and doctors in township hospitals (14.0%). In the 2021 survey, the most reference group chosen by rural doctors was the villagers (36.1%), followed by rural teachers (34.4%) and other village doctors (16.1%). It can be seen that rural teachers and villagers are the two major groups of rural doctors (Table 2).

**Table 2:** Selection of reference groups for rural doctors

Reference groups	2015(*N* = 307)		2021(*N* = 366)	
	*N*	%	*N*	%
Villagers	52	16.9	132	36.1
Other village doctors	20	6.5	59	16.1
Rural teachers	182	59.3	126	34.4
Village cadres	2	1.0	11	3.0
Township cadres	5	1.6	2	0.5
Doctors in township hospitals	43	14.0	33	9.0
Others	2	0.7	3	0.8

### Changes of village doctors’ sense of relative deprivation

#### Changes in vertical deprivation

Compared with 2015, the vertical deprivation score of rural doctors increased from 2.77 ± 0.81 to 3.04 ± 0.83 in 2021. The scores of vertical deprivation in social status, professional reputation and living standard increased from 2.87 ± 0.93, 2.34 ± 0.92 and 2.77 ± 1.12 in 2015 to 3.05 ± 0.94, 2.81 ± 0.87 and 3.08 ± 0.99 in 2021, respectively, with significant differences (*P* < 0.05). In terms of economic income, there was no significant difference between the two surveys (Table 3).

**Table 3:** Comparison of rural doctors’ vertical deprivation in the two surveys

Items	2015(±S)	2021(±S)	*t*	*P*
Total points	2.77 ± 0.81	3.04 ± 0.83	-4.11	<0.001
Economic income	3.13 ± 1.23	3.21 ± 1.09	-0.88	0.37
Social status	2.87 ± 0.93	3.05 ± 0.94	-2.51	0.01
Professional reputation	2.34 ± 0.92	2.81 ± 0.87	-6.79	<0.001
Living standard	2.77 ± 1.12	3.08 ± 0.99	-3.75	<0.001

### Changes in horizontal deprivation

#### Overall horizontal relative deprivation

Without considering the reference group, the horizontal relative deprivation scores of village doctors in the two surveys were 3.36 ± 0.87 and 3.45 ± 0.80, respectively, with no statistically significant difference. Among the four items, the relative deprivation score of professional reputation increased from 2.63 ± 1.03 in 2015 to 3.11 ± 0.95 in 2021, with a statistically significant difference (*P* < 0.01). There was no significant difference in economic income, social status and living standard (Table 4).

**Table 4:** Comparison of horizontal deprivation among village doctors in the two surveys

Items	2015(±S)	2021(±S)	*t*	*P*
**Overall horizontal relative deprivation**				
Total points	3.36 ± 0.87	3.45 ± 0.80	-1.42	0.16
Economic income	3.95 ± 1.15	3.82 ± 0.94	1.63	0.10
Social status	3.31 ± 1.02	3.31 ± 0.91	0.05	0.96
Professional reputation	2.63 ± 1.03	3.11 ± 0.95	-6.18	<0.01
Living standard	3.55 ± 1.13	3.57 ± 0.94	-0.33	0.74
**Horizontal deprivation compared with rural teachers**				
Total points	3.47 ± 0.87	3.97 ± 0.77	-5.26	<0.001
Economic income	4.12 ± 1.15	4.40 ± 0.81	-2.47	0.01
Social status	3.42 ± 1.00	3.87 ± 0.92	-4.06	<0.001
Professional reputation	2.68 ± 1.07	3.60 ± 1.01	-7.60	<0.001
Living standard	3.65 ± 1.15	4.02 ± 0.93	-3.11	<0.01
**Horizontal deprivation compared with villagers**				
Total points	3.09 ± 0.87	3.15 ± 0.57	-0.45	0.66
Economic income	3.69 ± 1.09	3.57 ± 0.79	0.75	0.46
Social status	2.96 ± 0.95	2.92 ± 0.66	0.26	0.80
Professional reputation	2.38 ± 0.93	2.68 ± 0.76	-2.05	0.04
Living standard	3.33 ± 1.12	3.42 ± 0.79	-0.58	0.58

### Horizontal deprivation of rural teachers as the reference group

In 2015 and 2021, 59.3% and 34.4% of village doctors chose rural teachers as the reference group. Overall, with rural teachers as the reference group, the horizontal deprivation scores of village doctors increased from 3.47 ± 0.87 in 2015 to 3.97 ± 0.77 in 2021, and the difference was statistically significant (*P* < 0.001). Specifically, the scores of economic income, social status, professional reputation and living standards deprivation increased significantly, and the differences were statistically significant (Table 4).

### Horizontal deprivation of villagers as the reference group

In 2015 and 2021, 16.9% and 36.1% of rural doctors chose villagers as the reference group. Overall, with villagers as the reference group, the horizontal deprivation scores of rural doctors in the two surveys were 3.09 ± 0.87 and 3.15 ± 0.57, respectively, with no significant difference. Specifically, the scores of professional reputation deprivation increased from 2.38 ± 0.93 to 2.68 ± 0.76, and the difference was statistically significant (*P* < 0.05). There was no significant difference in deprivation scores of economic income, social status and living standard (Table 4).

## Discussion

To the best of our knowledge, this is the first reproducible cross-sectional study to explore changes in the relative deprivation of rural doctors in the field of rural health care in China. The results of this study found that in the process of health care reform, rural doctors lost the vested interests under the previous system, the reference group in the rural social environment changed, and the vertical and horizontal relative deprivation was aggravated. This finding will help policy makers to understand the current practice mentality and development dilemma of rural doctors, which is conducive to stabilizing the team of rural doctors and ensuring the bottom of the rural health service network.

### The vertical relative deprivation of rural doctors is aggravated

Compared with 2015, the vertical relative deprivation of rural doctors increased in 2021, which was mainly caused by the increased deprivation of social status, professional reputation and living standard. The shortage of health care workers in rural and underdeveloped areas is a global challenge. Similar to the results of this study, Nasrin found that the factors of retaining rural health care personnel included financial, career and professional, working conditions, personal, cultural and living conditions factors through systematic review analysis [36].

The possible reasons are as follows: First, the workload of rural doctors is increasing. Since the new medical reform, rural doctors mainly undertake basic medical services and basic public health services. In 2017, the basic public health service projects were expanded from 41 projects in 9 categories to 55 projects in 14 categories [37]. This has led to an increasing workload for village doctors, and some studies have found that after the implementation of the project, the workload has increased to a level that village doctors cannot afford [38]. At the end of 2019, since the COVID-19 pandemic, village doctors have taken on a large amount of epidemic prevention and control work in rural areas, and their workload has further increased.

Second, the practice risk faced by village doctors has increased. On the one hand, the practice environment of rural doctors is poor, and the awareness and ability of medical safety are not strong, so medical disputes are prone to occur [39]. On the other hand, in the early stage of the COVID-19 epidemic, medical and health conditions in rural areas were weak, protective materials and means were inadequate, and rural doctors in the front line of the epidemic faced a greater risk of infection. During the pandemic, village doctors began to take on nucleic acid sampling, fever patient transport, key population management and other work, and the working environment had a high risk of infection [40].

Third, the income level of rural doctors has not improved significantly. After the implementation of the new medical reform, the main source of income for rural doctors is financial subsidies based on the service population [4]. Under this system, the income of rural doctors is stable, but the growth rate is slow, far from reaching their expected level, leading to the decrease of their sense of gain in income. In most studies, income level was the factor that influenced doctors’ reluctance to stay in rural areas to provide care [41, 42]. In a study of rural doctors in Japan, income dissatisfaction was significantly related to resignation intention. And the study showed that compared with the rural work burden, income satisfaction will be affected by personal perception of income value [43]. For a long time, rural doctors have been respected in the traditional rural society for their medical skills to meet the basic medical needs of rural residents. Under the combined effect of the increase of workload, the increase of professional risk and the lack of obvious improvement of income, rural doctors have a low self-evaluation of their professional reputation and social status [44, 45], and feel that their social status, professional reputation and living standards are not as good as before, resulting in a sense of vertical deprivation.

### The reference group of rural doctors has changed

The analysis results showed that rural teachers and villagers were the two main attribution groups of rural doctors, and the main reference group of village doctors has changed from rural teachers in 2015 to villagers in 2021. Since 1968, China has trained barefoot doctors to provide basic medical services to the rural population. In 1981, the State Council issued a policy document stipulating that barefoot doctors, as intellectuals in rural areas, should be treated equally with rural teachers [46]. Rural doctors choose rural teachers as their belonging group because they have certain historical similarities. They belong to the same rural intellectuals, who have relatively high educational level in rural areas and rely on their own knowledge and skills as their professional mental workers. They are similar in social status, economic income and professional reputation, and even rural doctors have more advantages [21]. However, with the advancement of rural education reform, rural teachers enjoy the salary and welfare benefits provided by the government. Rural doctors have not been guaranteed a salary by the government, and the gap between rural doctors and rural teachers in terms of economic income, professional reputation and social status has gradually widened, and it is difficult to make up [47]. As a result, rural doctors will no longer compare with teachers in the 2021 survey, but change the reference group to ordinary villagers.

However, with the change of the criteria for judging rural social status, rural doctors do not have much advantage over migrant workers and ordinary agricultural workers [45]. In addition, within the medical system, rural doctors and doctors in township hospitals are in the same rural social environment and serve the same people. Compared with doctors in township hospitals, rural doctors have a single income channel and lack of social security. Similarly, as grass-roots health workers, doctors in township hospitals have relatively good income level and job stability [48]. The results of a study in Turkey showed that the increase in doctors’ income reduced their willingness to work in rural areas [49]. Merton [10] for the first time clarified the concept of “reference group” in the sense of relative deprivation. Davies [50] and Yang [51] et al., further pointed out that the selection of “reference group” would affect individuals’ self-evaluation and their level of relative deprivation. In this study, the change of the reference group of rural doctors could partly reflect the decrease of their expected level and the enhancement of relative deprivation.

### The horizontal relative deprivation of rural doctors is enhanced

Village doctors have a strong sense of relative deprivation when compared with the reference objects, because they lost the vested interests under traditional social conditions and have not got the expected reform benefits, consistent with Peng’s study [52]. The horizontal relative deprivation of rural doctors reflects their dissatisfaction and unfairness compared with the reference group, and is the subjective judgment of their own deprivation. The results of our study have shown that, without considering the reference group, there is no significant change in the horizontal relative deprivation, only the deprivation of professional reputation is enhanced. In the case of considering the reference group, compared with the villagers, the sense of deprivation also increased significantly only in terms of professional reputation. However, compared with rural teachers, their overall horizontal relative sense of deprivation increased, and the sense of deprivation in economic income, social status, professional reputation and living standards increased significantly.

The possible reasons are as follows: First, the salary of rural doctors is relatively low. At present, the income sources of rural doctors include general medical fees, basic drug subsidies and basic public health service subsidies. Studies have estimated that the average monthly income of village doctors in 2019 was 2,421 yuan in China [53]. During the COVID-19 epidemic, the policy stipulated that village clinics could not receive patients with typical symptoms of COVID-19, such as fever and cough, while patients with these common and frequently-occurring diseases were the main patients in village clinics, which further reduced the income of village doctors [54]. The salary of rural teachers in China is mainly composed of basic salary, performance salary and subsidies. Liu’s research found that in 2020, the average monthly salary of rural teachers was 4,598 yuan in China [55], which was significantly higher than that of village doctors. Previous studies have also found that after the implementation of the essential medicine system, the average annual income of rural doctors has declined, significantly lower than that of rural teachers [56], and some village doctors even believed that their income was lower than that of villagers in the same rural [57]. Previous studies have shown that the income of rural doctors in India is unsatisfactory [42]. But in Japan, Matsumoto’s findings showed that rural doctors in Japan were so satisfied with their income that their salaries do not decrease even when financial losses occur [58].

Secondly, rural doctors have a higher work burden than rural teachers [59]. As the main health service providers in rural areas, rural doctors have been working almost all day and all year round for a long time. Rural teachers can not only enjoy statutory holidays, but also have winter and summer holidays, workload and work pressure are relatively small. Previous studies showed that the turnover intention of rural doctors was 46.9% [60], which was higher than that of rural teachers’ 26.7% [61].

Thirdly, the old-age security level of rural doctors is lower than that of rural teachers. Rural teachers belong to the career establishment, enjoy the old-age insurance for urban workers provided by the state, with a high level of old-age security. Rural doctors do not belong to public officials, but are essentially farmers [62], who can only participate in the old-age insurance for urban and rural residents purchased by themselves, and the level of security is low.

Fourthly, the practice risk faced by rural doctors is significantly higher than other groups. Rural doctors often face problems such as tense doctor-patient relationship and frequent medical disputes in their daily diagnosis and treatment activities [39]. During the COVID-19 pandemic, village doctors have been working on the front line of fighting the epidemic, and the risk of being infected is also very high. Rural teachers stopped offline teaching activities during the COVID-19 pandemic and taught through the form of online teaching, facing fewer disputes from parents and students. In addition, neither rural teachers nor other groups in rural areas need to participate in epidemic prevention and control work, and the risk of infection is relatively small compared with rural doctors. Studies have shown that low returns, heavy workload and high risk of practice can easily lead to the loss of professional pride and self-confidence of rural doctors [63], which leads to the lower professional reputation of village doctors than that of rural teachers and ordinary villagers, thus triggering a sense of horizontal relative deprivation.

#### Strengths and limitations

The advantage of this study is that previous studies have not paid enough attention to the relative deprivation of rural doctors. This is the first repeated cross-sectional study on the relative deprivation of rural doctors. At the same time, this study also has some limitations. Firstly, we should note the limitations of the cross-sectional study. This study is not a cohort study, and the lack of a stable sample of rural doctors may affect the generalizability of the results, so the representation of changes in rural doctors’ relative deprivation may be limited. Secondly, only two cities in Shandong Province were selected for the field survey, which may represent the rural doctors in Shandong Province to a certain extent, but the results of the study cannot represent the national level. Thirdly, this study did not systematically explore the influencing factors of rural doctors’ relative deprivation. In future research, we hope to complete a large national sample survey and further explore the influencing factors of relative deprivation.

## Conclusion

As the “bottom” of the three-level medical and health service network in rural China, rural doctors have become a channel to improve the quality of medical services in rural China under the serious shortage of health resources in rural areas. Rural doctors with a negative practice mentality may reduce the quality of health care provided to rural residents. The results of this study found that compared with 2015, village doctors surveyed in 2021 had a sense of vertical deprivation due to increased workload, increased practice risk and insignificant improvement in income level. The reference group selected by village doctors has changed from rural teachers to ordinary residents. Compared with rural teachers, village doctors have relatively low salary, high work burden, low pension security level, and the higher practice risk than other rural groups, thus resulting in a sense of horizontal deprivation. The sense of relative deprivation is likely to cause mental imbalance, which leads to the rationalization of village doctors’ disadvantages and has a negative impact on the quality of their work. If the interests of rural doctors are not solved for a long time, it may lead to social instability. This study further emphasizes the importance of rural doctors in the reform of rural health care in China, which will arouse the attention of the society to the group of rural doctors. Therefore, the government and relevant departments should establish policies suitable for rural society, let the dividend of medical reform benefit village doctors, reflect their social value, and mobilize their enthusiasm to provide medical and health services, so as to ensure the stability of rural health personnel.

## Data Availability

The datasets generated and analyzed during the current study are not publicly available due to data analysis has not been completed but are available from the corresponding author on reasonable request.
